# Is negative e-WOM more powerful? Multimodal data analysis on air passengers’ perception of COVID-19 safety measures

**DOI:** 10.3389/fpsyg.2022.983987

**Published:** 2022-10-18

**Authors:** Shizhen Bai, Dingyao Yu, Mu Yang, Rui Tang, Hao He, Jiayuan Zhao, Peihua Huang

**Affiliations:** ^1^School of Management, Harbin University of Commerce, Harbin, China; ^2^Department of Management, Birkbeck, University of London, London, United Kingdom; ^3^School of Economics Teaching and Research, Party School of the Central Committee of C.P.C (Chinese Academy of Governance), Beijing, China; ^4^School of Computer and Information Engineering, Harbin University of Commerce, Harbin, China

**Keywords:** e-WOM, negative safety perception, COVID-19 safety measures, multimodal reviews, sentiment analysis, review usefulness, airline choice

## Abstract

During the normalization stage of the COVID-19 epidemic prevention and control, the safety threats caused by improper epidemic prevention measures of airlines have become the primary concern for air passengers. Negative e-WOM related to safety perception obtained based on online multimodal reviews of travel websites has become an important decision-making basis for potential air passengers when making airline choices. This study aims to examine the relationship between potential air passengers’ negative safety perception and the usefulness of online reviews, as well as to test the moderating effect of review modality and airline type. It also further explores the effectiveness and feasibility of applying big data sentiment analysis to e-WOM management. To this end, the theoretical model of negative safety perception, review modality, and airline type affecting review usefulness was constructed. Then we select 10 low-cost airlines and 10 full-service airlines, respectively, according to the number of reviews sorted by the TripAdvisor website, and use crawling techniques to obtain 10,485 reviews related to COVID-19 safety of the above companies from December 2019 to date, and conduct safety perception sentiment analysis based on Python’s Textblob library. Finally, to avoid data overdispersion, the model is empirically analyzed by negative binomial regression using R software. The results indicate that (1) Negative safety perception significantly and negatively affects review usefulness, that is, extreme negative safety perception can provide higher review usefulness for potential air passengers. (2) Review modality and airline type have a significant moderating effect on the relationship between negative safety perception and review usefulness, in which multimodal reviews and full-service airlines both weakened the negative impact of negative safety perception on review usefulness. The theoretical model in this paper is both an extension of the application of big data sentiment analysis techniques and a beneficial supplement to current research findings of e-WOM, providing an important reference for potential air passengers to identify useful reviews accurately and thus reduce safety risks in online decision-making.

## Introduction

Advanced digital information technology has revolutionized traditional consumption channels (Khatwani and Srivastava, 2018). E-commerce platforms, mainly online shopping websites, have started to rapidly spread in various consumption scenarios such as retail, restaurants, hotels, and aviation, where consumers can readily share and exchange product information and shopping experiences through online reviews (Furner and Zinko, 2017; Akbari et al., 2022). Electronic word of mouth (e-WOM) is becoming the main source of product and service information for consumers and provides an important basis for consumers’ online shopping decisions (Lee et al., 2017; Ismagilova et al., 2020).

Based on the traditional word-of-mouth feedback mechanism (Zhang and Cole, 2016), e-WOM has broken through the limitations of time and space with the advancement of information technology. It can be regarded as an online customer feedback system that utilizes the two-way communication capabilities of the network, enabling individuals to share experiences and opinions related to companies, products, or services on the Internet (Brochado et al., 2019). The transmission of e-WOM has become a crucial marketing method, whose principle is essentially a process of signal transmission. As a signal, e-WOM moves from the sender (marketer or consumer) to the receiver (marketer or consumer). In the process of signal transmission, e-WOM established a trust relationship between individuals and firms (Akbari et al., 2022), thus affecting consumers’ purchase intention (Kumar and Singh, 2022). The latest research findings also reveal the effectiveness of e-WOM as a marketing method. For example，research has shown that e-WOM can enhance travelers’ willingness to pay for green hotels (Galati et al., 2021). And e-WOM can also stimulate the ethnocentrism of consumers and exert influence on the source selection of commodity brands (Sun et al., 2021).

Online reviews, as the main form of e-WOM (Lee et al., 2017; Mariani and Borghi, 2021), influence consumers’ shopping decisions through their usefulness. Review usefulness is interpreted as the perceived value of a specific product in making a purchase decision, i.e., the extent to which online reviews can help consumers make the right purchase decision (Mudambi and Schuff, 2010). Studies have shown that the usefulness of online reviews is related to the information volume of online reviews, but the emergence of a large number of online reviews with the help of mobile Internet will lead to review information overload (Park and Lee, 2008; Furner and Zinko, 2017), increasing the decision-making cost and risk of consumers (Singh et al., 2017). Big data technology can filter invalid review information and match consumers’ needs with useful online reviews to promote consumption. Based on the big data technology, exploring the influencing factors of review usefulness, identifying useful online reviews, and improving the quality of consumer decision-making have always been the major research motivation of e-WOM marketing (Kasabov, 2016).

Factors affecting the usefulness of online reviews are mainly measured by both quantitative and qualitative methods (Qazi et al., 2016). We attempt to conduct an inductive analysis of the factors influencing the usefulness of online reviews from the perspective of review content and reviewer characteristics. First, the content of online reviews affects review usefulness. Among them, review valence, review timeliness, review length, and the number of included pictures are quantitative factors(Racherla and Friske, 2012; Fang et al., 2016; Ma et al., 2020; Shi et al., 2020), which can be collected directly from online platforms. Review readability, review sentiment, review authenticity, commodity type, etc. are qualitative factors (Filieri, 2016; Chen et al., 2021; Qi et al., 2021), which cannot be obtained directly, and require rigorous quantitative methods to translate into quantitative factors. Second, characteristics of online reviewers, such as reviewer reputation, reviewer professionalism, reviewer experience, etc. also affect review usefulness (Park and Kim, 2008; Racherla and Friske, 2012). This paper uses both quantitative and qualitative factors to innovate the research in the usefulness of online reviews, and relies on text mining and big data sentiment analysis technology to quantify the qualitative data.

Based on a review of the literature related to the influencing factors of the usefulness of online reviews, we can identify at least three research gaps (Purnawirawan et al., 2012; Liu and Park, 2015; Leung, 2021). First, the current research scenarios on the influencing factors of online review usefulness mainly focus on the hospitality and catering industries (Li et al., 2020; Leung, 2021), and rarely involve the aviation industry. In fact, it is significant and essential to explore the factors that influence the usefulness of online reviews of airlines in the aviation industry. The airline choice of potential air passengers relies heavily on online travel platforms’ online reviews (Ho and Lee, 2007; Chong and Law, 2019), and useful online reviews can reduce the information asymmetry between air passengers and airlines. The airlines’ e-WOM is not only an important decision-making basis for air passengers to choose an airline, but also an important channel for airlines to collect air passengers’ feedback information (Zhang and Cole, 2016). Especially during the epidemic, the aviation industry has been severely impacted, and feedback information from passengers’ online reviews is a crucial reference for promoting the recovery of airline performance.

Second, the vast majority of studies on the usefulness of online reviews in the aviation industry focus on factors such as price, service quality, and satisfaction (Brochado et al., 2019; Sezgen et al., 2019), but current scholars rarely consider the impact of specific content related to COVID-19 safety contained in online reviews on the usefulness of reviews. Not every airline has been able to take safe and effective epidemic prevention and control measures since the December 2019 outbreak of the COVID-19, and the risk of cross-border and in-flight transmission of the virus remains (Bielecki et al., 2021). Due to the fear of contracting the virus, safety has become the top priority for air passengers when choosing airlines to travel. Studies have shown that online reviews generated by users are more credible than the information disclosed by official websites or other channels, so potential air passengers are more inclined to perceive whether the airline’s epidemic prevention and control measures are safe and effective from online reviews (Davras and Durgun, 2021). In this case, online reviews related to COVID-19 safety measures are more helpful for air passengers to make safe travel decisions. However, there is no research has been conducted on how airline online reviews containing safety perception information affect review usefulness in the context of an epidemic.

Finally, few scholars have explored how negative e-WOM associated with safety perception affects the usefulness of airlines’ online reviews from a specific emotional perspective. It has been demonstrated that the sentiments conveyed by online reviews can significantly affect the usefulness of online reviews (Ahmad and Laroche, 2016), but previous studies focused more on the sentimental inclination and intensity of reviews (Chua and Banerjee, 2016), lacking a separate consideration of negative sentiment. Studies have shown that in order to fully understand and avoid potential risks and losses, people are more willing to believe negative reviews than positive and neutral reviews (Berezina et al., 2016). And in the research of the aviation industry, sentiment analysis has almost always been used in the fields of service quality and passenger satisfaction (Misopoulos et al., 2014; Siering et al., 2018; Xu et al., 2019). But how potential air passengers’ negative safety perception from browsing online reviews affects the usefulness of online reviews is still in the blank of research.

This paper will reflect the innovation and necessity of this research by filling the above three research gaps. We seek to extend the study of the usefulness of online reviews to the airline industry. In the context of the normalization of the epidemic, this paper focuses on potential air passengers’ negative perception of epidemic safety measures and explores how this perception affects review usefulness and how it further affects air passengers’ airline choices. The main source of negative safety perception is online reviews, so the modality of online reviews will be an important factor affecting the research results. Different types of airlines have different groups of passengers, and even in the face of the same online reviews, passengers who do not belong to different groups have different degrees of negative safety perception, which will also have an impact on the results of the study. Therefore, in order to make the research more rigorous and in-depth, the influence of “review modality” (single-modal versus multimodal) and “airline type” (low-cost airline and full-service airline) should also be paid attention to. Specifically, we mainly focus on two issues:

Question 1: How does the negative safety perception that potential air passengers receive through airline online reviews affect the usefulness of online reviews?

Question 2: How do airline type and review modality affect the relationship between potential air passengers’ negative safety perception and online review usefulness?

This study constructs a theoretical model to explore the influencing factors of online review usefulness, which accurately reveals the complex relationship between safety perception, review sentiment, airline type, review modality and online review usefulness. The study innovates the research scenario of e-WOM and develops influencing factors of online review usefulness from the perspective of air passengers’ safety concerns. In addition, the big data sentiment analysis techniques are used to enrich the theoretical basis for the behavior pattern of air passengers’ airline choices by identifying useful online reviews. More importantly, helping potential air passengers more effectively identify useful online reviews can help reduce the safety risks of choosing an airline to travel during the pandemic.

Therefore, the structure of this paper is organized as follows: the literature review section first reviews earlier research findings related to the usefulness of online review, negative safety perception, airline type, and review modality, then constructs a theoretical model and formulates research hypotheses based on the model. The methodology section describes the selected samples, data collection and processing, and variable calculation. The analysis section reveals the analysis methods, analysis process, and analysis results. Finally, the research findings and implications, limitations, and future research directions are summarized in the conclusion and discussion section.

## Literature review and hypotheses development

### Theoretical background and conceptual framework

Protection motivation theory suggests that when people face the threat of certain risk factors, they will adopt certain risk-averse behaviors out of the need to protect their own interests (Wang et al., 2019). It reveals people’s cognitive processes of risk assessment and risk response through “threat appraisal” and “coping appraisal” (Shafiei and Maleksaeidi, 2020; Kim et al., 2022). “Threat appraisal” reveals the severity of the threat and the likelihood of being threatened, and “coping appraisal” reveals the effectiveness of the response to the threat and the ability to implement the response to the threat (Tsai et al., 2016).

During the epidemic, a series of behavioral patterns of potential air passengers are guided by the protective motivation theory. Potential air passengers first measure the virus threat they face during travel on the basis of the severity of the current epidemic and the possibility of contracting the virus, which is essentially a process of “threat appraisal.” After a full assessment of the virus threat, air passengers begin to take a series of preventive and protective measures to safeguard their health and safety. For example, they try to identify useful online reviews according to negative safety perception, and then judge whether the airline’s epidemic prevention and control measures are safe and effective to choose the safest airline to reduce the risk of contracting the virus while flying. And this is actually a process of “coping appraisal.” Therefore, the protection motivation theory reveals the motivation and behavioral process of air passengers choosing the safest airline through online reviews, which provides precise theoretical support for our research framework.

Prospect theory argues that people are asymmetrically sensitive to loss and gain, and the pain of facing loss far outweighs the pleasure of facing gain (Barberis, 2013). Most people prefer risk aversion when faced with benefit gain (Nicolau, 2011), especially when it comes to life safety. Prospect theory reveals why air passengers prefer to browse and believe negative online reviews. In order to understand the true effectiveness of the airline’s epidemic prevention and reduce the risk of virus infection due to uncertainty, potential air passengers will pay more attention to the negative sentiment conveyed by online reviews related to epidemic safety measures, which also explains why negative reviews are more useful.

**Figure 1:**
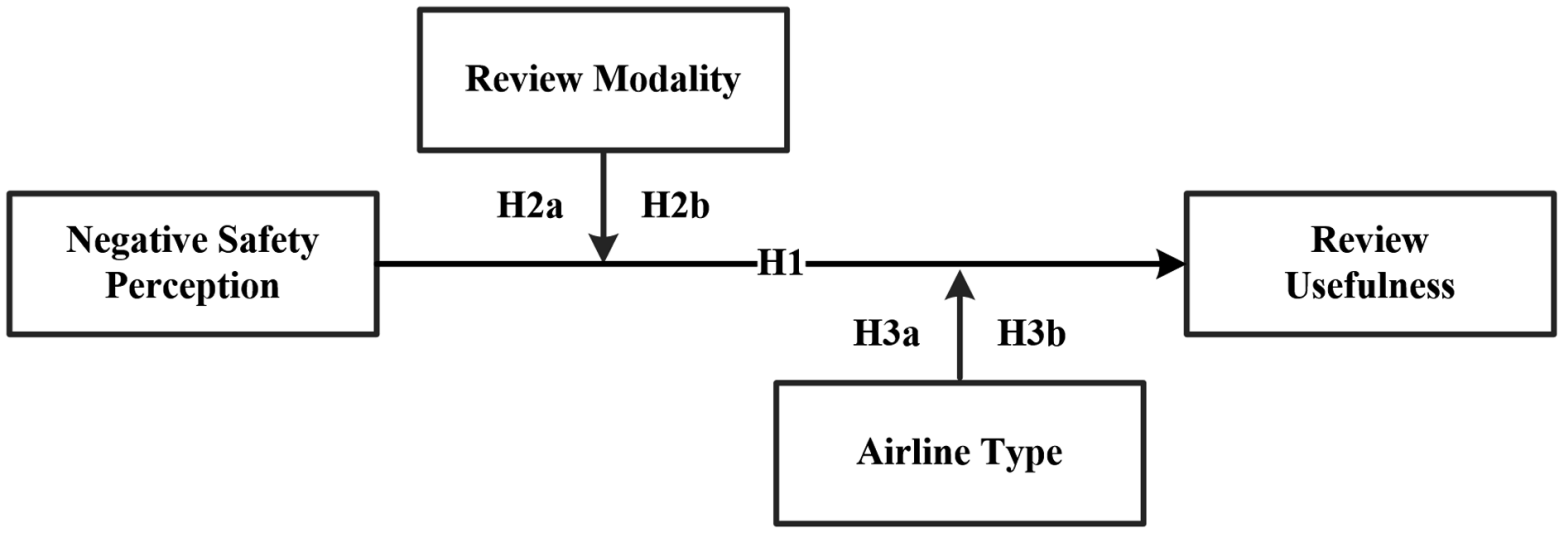
The conceptual framework.

Guided by protection motivation theory and prospect theory, an attempt was made to construct a conceptual framework for the study to answer the above two questions. As shown in Figure 1, the first objective of this study is to examine the relationship between potential air passengers’ negative safety perception and the usefulness of online reviews; the second objective is to explore whether the modality of online reviews and the type of airline will moderate the effect of negative safety perception on the usefulness of online reviews, and if so, how? The conceptual framework of the research guides the formulation of research hypotheses.

### Safety perception

Safety has always been the most concerned issue for air passengers (Yang et al., 2018), and it is also an important basis for choosing an airline (Fleischer et al., 2015). In past studies, safety perception, as a significant dimension to measure airline service quality, improve airline image, or evaluate air passenger satisfaction, had been widely used to predict airline selection decisions (Tsaur et al., 2002; Jou et al., 2008; Jeeradist et al., 2016). However, at different times, air passengers have different perception of airline safety. The turning event that gave rise to different safety perception is the outbreak of the COVID-19 epidemic in December 2019, before which the predominant safety threat was aircraft crashes, although there were cases of epidemic transmission (Yang et al., 2018), after the start of the pandemic, the risk of contracting the virus became a major component of air passengers’ safety perception (Bielecki et al., 2021).

The convenience and accessibility of air travel have accelerated the spread of the coronavirus globally (Sotomayor-Castillo et al., 2021; Sulu et al., 2022). Data from the International Air Transport Association (IATA) indicates that more than 4.5 billion air passengers departed worldwide in 2019. However, there is no international consensus on quarantine measures among different countries, so the effectiveness of epidemic prevention and control varies from country to country (Bielecki et al., 2021), which undoubtedly increases the possibility of air passengers contracting the virus. Airline bans are seen as an effective way to limit population mobility and reduce the speed of virus transmission, but the resulting side effects have plunged numerous airlines into financial crisis (Bielecki et al., 2021; Sulu et al., 2022), even leading to the inability to afford the huge costs required to implement effective epidemic prevention and control measures (Mouchtouri et al., 2019), which makes the safety effectiveness of measures implemented by each airline, especially low-cost airlines, vary greatly, further exacerbates the safety risks of air travel.

Air passengers’ perception of safety is essentially an assessment of safety concerns, which are considered to be an emotional experience consisting of a complex overlay of emotions such as anxiety, worry, and fear (Hosany and Gilbert, 2010; Rittichainuwat, 2013). Based on the above analysis, it is reasonable to assume that air passengers’ safety concerns are related to the uncertainty threat of perceived environmental safety. Fear of contracting the virus is the main emotion when facing the uncertainty threat (Cisler et al., 2009), and the threat of fear and uncertainty exacerbates air passengers’ psychological stress (Mamun, 2021), making them extra cautious when choosing an airline because they cannot be sure that the airline they choose can be proved to be safe, which severely hinders air passengers’ willingness to travel (Cahyanto et al., 2016). According to a recent survey of air passengers conducted by IATA, about 30% of passengers said they were not going to travel by air within 6 months (Bhuvan et al., 2021). This effect is not only reflected in the choice of airline. Due to the uncertainty of safety perception, people begin to deliberately maintain a safe social distance to avoid the risk of infection caused by crowding (Cao and Song, 2022), and begin to reduce unnecessary travel to reduce the likelihood of being infected by the virus during travel (Nazneen et al., 2022).

### Negative safety perception and online review usefulness

Much of the uncertainty in air passengers’ perception of airline safety is due to the fact that there is asymmetric safety information in the airline industry (Fleischer et al., 2015). Although many airlines will disclose the epidemic prevention and control measures they have taken in the context of an epidemic, air passengers are not able to judge whether these measures disclosed by airlines are really safe and effective due to the existence of the “lemon market” (Nicolau and Sellers, 2010). Airline safety services, as experiential goods, can only be determined through consumption (Mudambi and Schuff, 2010), so the uncertainty of safety perception cannot be eliminated in advance.

The uncertainty reduction theory holds that in order to obtain more decisive information about products and services, consumers will actively explore external objective information to reduce uncertainty (Lee et al., 2017). Compared with controlled information sources like official disclosures, user-generated information presented in online reviews is more credible (Davras and Durgun, 2021),and has greater perceived certainty. Brochado et al. (2019) points out that air passengers consider e-WOM shared by other passengers to be trustworthy. Therefore, the online review information of third-party travel platforms provides air passengers with a new channel to perceive the airline’s true safety level and is also an important source of information for passengers to choose safe airlines.

Sparks and Browning (2011) argue that in the decision-making process, online reviews can provide useful information to customers through the content of the reviews and the sentiments conveyed by the reviews. It is the usefulness of such reviews that helps customers mitigate their perception of purchase risk and uncertainty before engaging in the service experience (Cantallops and Salvi, 2014). Based on the conclusions of this study, we can repute that considering both the specific review content related to the research topic and the specific sentiments conveyed by online reviews can make online reviews more useful for decision-making support.

Specifically, online reviews that are strongly related to safety-perception content will be more useful. Because consumers nowadays hope to quickly retrieve review information that is particularly useful for their specific purchase decisions (Filieri et al., 2021). Such reviews contain real evaluations of the safety effectiveness of airline epidemic measures provided by other air passengers, which is convenient for potential air passengers to obtain more accurate safety perception to support their airline choice decisions.

In addition to this, online reviews that convey a particular sentiment (positive, negative, or neutral) would be more useful because this particular sentiment influences consumers’ purchase intentions (Wang et al., 2017). Based on the loss-avoidance properties of prospect theory, consumers are more sensitive to negative reviews (Van Noort et al., 2007) and are more inclined to search for and believe negative reviews. Negative reviews provide more diagnostic information than neutral or positive reviews (Berezina et al., 2016), so negative reviews are considered more useful, and the more extreme the negative sentiments in the review, the more useful the review will be, as reviews with extreme sentiments are considered more persuasive (Fang et al., 2016). Although there are also studies that put forward different points, for example, Mudambi and Schuff (2010) believe that for experiential products, neutral reviews are more useful than positive or negative reviews. Cai et al. (2017) proves that review usefulness is related to the intensity of negative emotions, strong negative emotions reduce the usefulness of negative reviews, while moderate negative emotions can increase the usefulness of negative reviews. We still believe that the safety perception obtained by air passengers through negative online reviews related to epidemic safety, that is, negative safety perception, can help air passengers make correct airline selection decisions, and the more negative the safety perception, the more useful the online reviews. Accordingly, we propose Hypothesis 1:

*H1*: Negative safety perception has a significantly negative influence on review usefulness, i.e., extreme negative safety perception provides potential air passengers with higher review usefulness.

### The moderating effect of review modality

People’s information exchange and emotional expression usually use various data modalities (Ma et al., 2020), which refer to the different channels of information that are semantically correlated and provide complementary information to each other (Razzaghi et al., 2021), such as text, audio, pictures and videos. According to the number of data modalities included, it can be divided into single-modal data and multimodal data. Multimodal data is defined by the presence of more than one modality or channel, e.g., visual, audio, text, gestures, and eye gage (Poria et al., 2017). Online reviews as a kind of big data for social media communication, text and pictures are the two most common modalities. Therefore, it can also be divided into single-modal reviews (text only) and multimodal reviews (i.e., reviews with both text and pictures) according to the data modalities contained (Xiao et al., 2022).

With the gradual enrichment of online feedback functions in tourism websites, user-generated e-WOM gradually shifted from a single modality of text to multimodalities. To express richer information, air passengers prefer to use multimodal online reviews to share their opinions and experiences about airlines. Past research on online reviews has mainly focused on the text mining and analysis of reviews, ignoring the sentiment tendencies of consumers after viewing the picture information in online reviews, and only considering “whether to upload pictures” (Shi et al., 2020), “the number of pictures included” (Zhang and Li, 2016) as a factor that affects the usefulness of reviews in the review content, but failed to compare and evaluate the difference in the impact of multimodal reviews and single-modal reviews on the usefulness of online reviews from the perspective of review modality. We attempt to clarify how different review modalities moderate the relationship between air passengers’ negative safety perception and the usefulness of online reviews. Specifically, we test the moderating effect of review modality from the perspectives of information processing and sentiment recognition.

From the perspective of information processing, according to the dual coding theory of cognitive psychology (Paivio, 2014), people have two independent information processing systems, the verbal system that reveals textual information and the nonverbal system that reveals visual information. When encoding both textual and visual information, the verbal and nonverbal systems will be connected (Paivio, 2014), and the textual information and visual information will have complementary effects, thereby improving the information processing effect and making multimodal information that combines pictures and text easier to understand and remember than single-modal information. Pictures and texts in airline online reviews have certain semantic correspondences, which can complement each other’s lack of semantic information (Wang and Chen, 2018), helping air passengers obtain more accurate perception of safety. It has also been argued that picture information in multimodal online reviews adds diagnostic value to textual information and reduces perceptual uncertainty, thus increasing the usefulness of multimodal online reviews (Wu et al., 2021). In addition, multimodal reviews containing pictures are more persuasive than textual reviews because they can attract more viewers’ attention (Zhang and Li, 2016), which is also considered to be one of the reasons that make multimodal online reviews more useful. It can be seen that multimodal online reviews can improve the usefulness of online reviews.

From the perspective of sentiment recognition, pictures in multimodal online reviews have the same function of conveying emotion as text, and the correlation and complementarity between different modal information make the effect of sentiment classification more objective and accurate (Xie et al., 2021). The rich sentiment cues contained in the pictures of airline online reviews can corroborate and complement the sentiment information in the text, and eliminate the sentiment differences between different modal information to avoid the extreme sentiment misleading of single-modal false reviews, which helps improve the accuracy of air passenger negative safety perception recognition (Zhao et al., 2019; Zhang et al., 2021). Therefore, we believe that multimodal online reviews contribute to the accurate identification of sentiment.

Taken together, we suggest that the modality of online reviews may moderate the relationship between negative safety perception and online review usefulness and that multimodal reviews may reinforce the effect of air passengers’ negative safety perception on online review usefulness， whereby we propose hypothesis 2 to test the moderating effect of the online review modality:

*H2a*: Online review modality significantly moderates the relationship between negative safety perception and review usefulness.

*H2b*: Compared to single-modal reviews, multimodal reviews negatively moderate the relationship between negative safety perception and review usefulness, i.e., multimodal reviews reinforce the negative effect of negative safety perception on review usefulness.

### The moderating role of airline type

The epidemic prevention and control measures taken by airlines are essentially a safety service for air passengers during the COVID-19 period. Airline service quality affects air passenger satisfaction (Xu et al., 2019), and air passenger satisfaction is considered an important basis for airline selection. Especially during the epidemic, whether airlines can provide satisfactory safety services will significantly affect the airline selection decisions of air passengers.

Different types of airlines provide different quality safety services. Low-cost airlines attract passengers through cost-effectiveness, while full-service airlines provide value through high-quality safety services (Rajaguru, 2016). Therefore, for passengers who choose low-cost airlines, price is more important than perceived safety service quality (Kim and Lee, 2011). Customers who choose full-service airlines pay higher prices, according to the fair claim of social exchange theory (Emerson, 1976), it is obvious that They will demand a better safety service experience (Xu et al., 2019). It can be seen that the safety service quality expectations of air passengers for full-service airlines are usually higher than those of low-cost airlines. In other words, air passengers are more inclined to believe that during the epidemic, full-service airlines will take more effective safety protection measures than low-cost airlines and provide better safety services. Even if service failure occurs, full-service airlines will take more timely and effective safety service remedial measures than low-cost airlines (Xu et al., 2019). Since the quality of safety services of low-cost airlines and full-service airlines is considered to be different, air passengers have to consider the influence of airline type when choosing an airline. Online reviews that include airline type will provide air passengers with more useful information, and opting for full-service airlines mitigate the degree of negativity of air passengers’ safety perception.

In summary, we propose that airline type may moderate the relationship between negative safety perception and online review usefulness and that full-service airlines may mitigate the effect of air passengers’ negative safety perception on online review usefulness, Accordingly, we propose Hypothesis 3 to test the moderating effect of airline type:

*H3a*: Airline type significantly moderates the relationship between negative safety perception and review usefulness.

*H3b*: Compared with low-cost airlines, full-service airlines positively moderate the relationship between negative safety perception and review usefulness, i.e., full-service airlines weaken the negative impact of negative safety perception on review usefulness.

## Materials and methods

### Sample and data collection

The online review data of airlines used in this study comes from the TripAdvisor website. The reasons for choosing it as the data source are as follows: First of all, TripAdvisor, as the world’s largest online travel e-WOM platform (Mary and Pour, 2022), allows air passengers to make real-time online evaluations of most airlines in the world (Litvin et al., 2018) and disseminates reviews to potential air passengers (Taecharungroj and Mathayomchan, 2019). Secondly, according to the official website, the total cumulative number of reviews on TripAdvisor has exceeded one billion and it is still being added at a rate of over 200,000 reviews per day (Chen et al., 2021). A large amount of user-generated content provides sufficient real data to support this study (Chang et al., 2019). Finally, in addition to text reviews the TripAdvisor website also allows air passengers to post and share multimodal (pictures, emojis, etc.) information about their air travel experience, which makes it possible to carry out multimodal big data analysis for this study. Existing studies have shown that the number of online customer reviews can generate a large impact on consumers’ purchase decisions (Cheng and Ho, 2015). Therefore, in order to determine the best airline samples, we develop the first web crawler based on Python to help us obtain all airline names and the corresponding cumulative number of English-language reviews on TripAdvisor as of April 17, 2022. We distinguish between low-cost airlines and full-service airlines and select the top 10 airlines, respectively, according to the order of the cumulative number of English reviews as our samples.

Then we develop a second automated data crawler to capture online reviews of 20 airlines on TripAdvisor. Referring to the official outbreak time of COVID-19 in December 2019 (Zhou et al., 2020), we set the data crawling time range from December 1, 2019, to April 17, 2022, with the aim of collecting reviews since the December 2019 outbreak to date. We collect a total of 61,436 online reviews from 20 sample companies. Since not all the sample data obtained are related to the topic of “epidemic safety,” keywords are set to filter the reviews directly related to “epidemic safety” from the sample data. Unlike the subjective keyword setting methods in existing studies (Davras and Durgun, 2021; Mary and Pour, 2022), for the purpose of maximizing the number of valid sample data, we use Python to conduct word frequency statistics on 61,436 reviews, from which high-frequency words directly related to “epidemic safety” are selected as screening keywords, such as “covid,” “safety,” “pandemic,” “coronavirus “, “mask,” “quarantine,” “vaccine,” etc. After that, the filtered reviews are de-duplicated, which in turn yielded 11,398 valid reviews directly related to the study topic.

**Figure 2:**
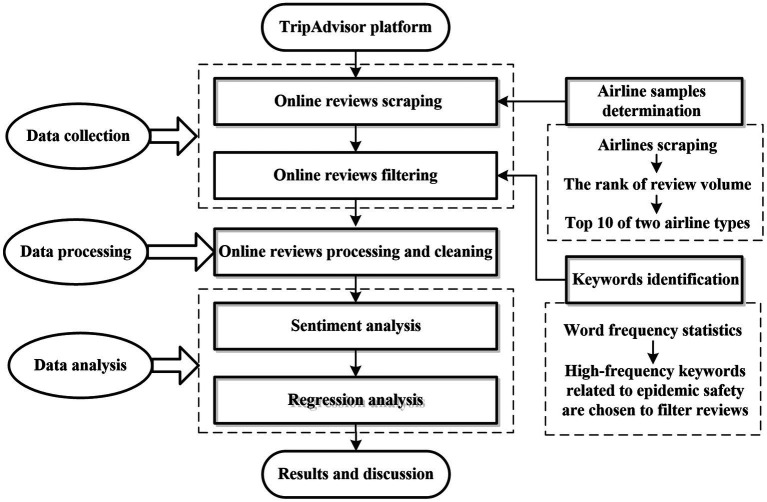
Data analysis process framework.

Finally, the valid reviews are cleaned and preprocessed, shorter reviews with less than five words and reviews other than English are excluded, and reviews with missing information and abnormalities are also deleted (Lai et al., 2021). Only 10,485 online reviews are retained for further sentiment analysis and regression analysis. Table 1 summarizes the sample and data collection, and Figure 2 shows the overall framework of the data analysis process.

**Table 1:** Summary of the sample and data.

**Selected sample airlines**	
Full-service airlines	American Airlines, Delta Air Lines, British Airways United Airlines, Emirates, Qatar Airways, Qantas Singapore Airlines, Lufthansa, Turkish Airlines
Low-cost airlines	Southwest Airlines, Ryanair, easyJet, Jet2.com Spirit Airlines, Air Canada, JetBlue, Norwegian TUI Airways (United Kingdom), Frontier Airlines
**Screening keywords**	
Related to “epidemic safety”	“covid,” “safety,” “pandemic,”"coronavirus,” “mask,” “quarantine,” “vaccine,” etc.
**Online reviews**	**Number**
Scraped online reviews of airlines during the epidemic	61,436
Online reviews filtered by keywords	11,398
Online reviews after treatment and cleaning	10,485

### Variables and measures

The usefulness of airline online reviews was used as the dependent variable. The quantitative measurement methods of review usefulness in existing research mainly focus on the following: (1) Scale scoring method (Wang and Chen, 2018); (2) Calculating the number of useful votes of reviews (Liu and Park, 2015; Fang et al., 2016); (3) Calculating the proportion of useful votes of reviews to the total number of votes (Mudambi and Schuff, 2010); (4) Calculating the useful vote ratio more precisely using Bayesian mean score methods (Qiu et al., 2019); (5) Calculating the cosine similarity of the vector of each review to the vector of all useful reviews as a proxy variable for the usefulness of the reviews using a machine learning algorithm (Shi et al., 2020). We analyzed the above methods. First, the scale scoring method is widely used in online marketing research, but the quality of the questionnaire data collected may be reduced due to factors such as the design flaws of questionnaires and the uncontrolled execution process (Denscombe, 2009; Jaeger and Cardello, 2022). Secondly, readers browsing reviews on TripAdvisor can vote on whether a review is useful, but the useful vote ratio is not available. Thirdly, calculating the cosine similarity of the vector ignores the reviewer’s context to some extent (Xiang et al., 2017). Therefore, we use the number of useful votes of reviews, which is commonly used in current research, as a proxy variable for the usefulness of reviews.

The independent variable is the negative safety perception of potential air passengers. Negative safety perception is the negative perception of potential air passengers about the safety of airline epidemic prevention and control formed after browsing online reviews. In order to investigate how the negative e-WOM related to “epidemic safety” affects the usefulness of online reviews, it is necessary to quantify the negative safety perception of potential air passengers.

Based on Natural Language Processing (NLP) and Computer-Aided Text Analysis (CATA) methods, we use Python’s Textblob library to perform sentiment analysis on online reviews collected from TripAdvisor websites to calculate the safety perception sentiment scores based on the online review text, thereby classifying and quantifying the sentiment polarity of each review, which has been shown to be an effective approach (Gauba et al., 2017). Textblob is a library developed based on Natural Language Toolkit (NLTK) and Pattern for processing English text, providing an easier-to-use API execution interface for some natural language processing tasks, such as lexical annotation, and noun phrase recognition, sentiment analysis, text classification, translation, etc. (Micu et al., 2017). Textblob’s text sentiment score calculation is essentially a dictionary-based implementation (Zhang and Cheng, 2021). Each online review is composed of several words and each specific identifiable word has its corresponding sentiment score according to the contextual relationship, lexicality of the word, sentiment strength, and position in the sentence (Bravo-Marquez et al., 2016), the sentiment score of each text review is calculated from the sentiment scores of the constituent words. The safety perception sentiment scores output by Textblob range from −1 to 1, with negative numbers indicating negative sentiment, 0 denoting neutral, and positive numbers representing positive sentiment, where −1 and 1 indicate extremely negative and extremely positive, respectively (Alabrah et al., 2022). Since only negative sentiment is considered in this study, after calculating the safety perception sentiment scores for the 10,485 online reviews, only 3,124 of them with negative sentiment scores are selected as independent variables for this study. Table 2 shows some examples of safety perception sentiment scores.

**Table 2:** Examples of safety perception sentiment scores.

**Online reviews related to COVID safety**	**Safety Perception Sentiment Scores**
Completely disappointed with how unclean this flight is, COVID cleanliness IS NOT happening with American Airlines	−0.7500
The plane was packed and I felt truly unsafe packed in this plane during a surge in covid truly disappointing	−0.6000
Flight only one-third full yet everyone herded together in rows of three, middle section empty and they would not allow anyone to move seats, hardly helping COVID measures yet you have to keep your mask on, Easyjet has no problems with this.	−0.0069
I was expecting the safety measures during the pandemic, but the seats were all full. We were served a simple sandwich that was not fresh and tasty. The flights were on time and the crew was hospitable.	0.0667
Excellent service very organized with the safety protocols for the covid-19. everyone should do their part n we will be safe.	0.5667
It’s always a pleasure to fly with EasyJet and I’m happy to see that they are doing their best to provide a safe environment during the pandemic.	0.6833

We also introduce two moderating variables, review modality and airline type. The first moderating variable is review modality. The rapid development of social media has gradually transformed the data types of online reviews from single-modal to multimodal. Compared with single-modal reviews, multimodal reviews rely on the information complementation mechanism between different modalities to improve the information perception ability of review readers, which helps us to accurately identify the real emotions of review readers (Poria et al., 2017). The online review data collected from TripAdvisor can be divided into two types: single-modal text data and multimodal data combining graphics and text. Therefore, we constructed a dichotomous dummy variable to quantify review modality and analyze the moderating effect of review modality on the main effects. The value is 0 if the review contains only text, and 1 if the review contains pictures in addition to text.

The second moderating variable is airline type. Airline type is an important factor influencing air passengers’ e-WOM and travel choices (Yang and Mundel, 2022). Existing studies suggest that air passengers’ travel choice is influenced by the perception of the value offered by the airline (Kim, 2015). Therefore, it is necessary to investigate the impact of air passengers’ safety perception on travel choices for different types of airlines based on online review data during the epidemic. Similar to the quantification method for review modalities, we construct dichotomous dummy variables, taking a value of 0 for low-cost airlines and a value of 1 for full-service airlines.

Finally, two control variables are added to avoid potential confounding effects. Referring to previous studies, combined sample data scraped, selecting control variables from the two dimensions of reviewer and review content will be more comprehensive (Lee et al., 2017; Chen et al., 2021). From the reviewer dimension, the reviewer expertise is critical, which can be reflected to a certain extent by the total number of comments published (Han, 2021). So we chose reviewer expertise as one of the control variables, measured by the total number of reviews posted by reviewers on TripAdvisor, which is a non-negative count variable. From the dimension of review content, we quantify the review valence of each review as another control variable by the star rating given by the reviewer, using a 5-point scale, where 1 means very disliked and 5 means very liked.

The specific variables are designed and measured as shown in Table 3. Most of these variables are available directly on the customer reviews page of the TripAdvisor platform.

**Table 3:** Summary of variables design and measures.

**Variable**		**Short**	**Operationalization**	**Notes**
DV	Review Usefulness	RU	Number of usefulness votes	Count variable
IV	Negative Safety Perception	NSP	Negative sentiment score of COVID safety perceived from online reviews measured by Textblob library	Range: [−1, 0)
MV	Review Modality	RM	Single-modality (=0) VS. Multi-modality (=1)	Virtual variable
	Airline Type	AT	Low-cost airlines (=0) VS. Full-service airlines (=1)	Virtual variable
CV	Review Valence	RV	The star rating of a review	Range: [1,5]
	Reviewer Expertise	RE	Number of online reviews written by the online reviewer	Count variable

DV: Dependent variable; IV: Independent variable; MV: Moderating variable CV: Control variable.

## Analysis and results

The results of descriptive statistics of variables are shown in Table 4. Among them, the mean value of review usefulness is 1.4632, which means that each review received at least 1 usefulness vote on average, of which 0 votes account for 20.77%, 1 votes account for 39.56%, 2 votes account for 24.49%, 3 votes account for 9.31%, and the part with more than 3 votes takes the rest 5.87%, which obviously obey the right-skewed distribution. This is due to the fact that the selected samples are all concentrated in the last 3 years and there are more restrictive conditions for screening the sample data. The mean value of negative safety perception is −0.1160, and more than 90% of the negative safety perception scores are concentrated between −0.5 and 0, which indicates that air passengers’ perception of epidemic safety is mostly moderately negative and mildly negative. The mean value of review modality is 0.1269, indicating that 12.69% of the reviews in the sample data contain both text and picture modalities. This percentage is significantly lower compared to the multimodal review percentage of hotel reviews, restaurant reviews, and e-commerce platform reviews, mainly because air passengers are required not to use their phones during the flight. The average value of airline type is 0.5477, which means that 54.77% of our final review sample data are from full-service airlines, and the remaining 45.23% are from low-cost airlines, which is well represented. This ratio shows that our final review data does not lose its representativeness due to the reasons from different types of airlines. If the final review data source is mainly concentrated on a specific type of airline, the regression analysis will be inaccurate. Since the only negative sentiment is considered in the sample data, a mean value of 1.8832 for review valence is normal. The mean and standard deviation of the reviewer’s expertise are strongly influenced by extreme values, so we standardize this variable by taking logarithms (Toothmaker, 1994).

**Table 4:** Descriptive statistics.

**Variable**	**Obs**	**Mean**	**Std. Dev.**	**Min**	**Max**
Review Usefulness	3,124	1.4632	1.7739	0	73
Negative Safety Perception	3,124	−0.1160	0.1188	−1	0
Review Modality	3,124	0.1269	0.1618	0	1
Airline Type	3,124	0.5477	0.4978	0	1
Review Valence	3,124	1.8832	1.4242	1	5
Reviewer Expertise	3,124	705.0407	8726.9330	1	197,139

The correlation coefficient matrix between the variables is shown in Table 5. None of the correlation coefficients among the variables exceeded 0.2619, which is far less than 0.5, indicating a low correlation among the variables. But to more accurately test for multicollinearity that might affect the regression analysis, we calculate the variance inflation factor (VIF) and tolerance (1/VIF) of the variables. Table 6 shows that all VIF values are distributed between 1 and 1.07, well below the commonly determined threshold of 10, and the tolerances are all greater than 0.1. Therefore, based on the results of the multicollinearity tests in Tables 5, 6, we can determine that our findings are not significantly affected by the multicollinearity problem.

**Table 5:** Correlations analysis between the variables.

**Variables**	**1**	**2**	**3**	**4**	**5**	**6**
Review Usefulness	1					
Negative Safety Perception	0.0250	1				
Review Modality	0.1005	0.0348	1			
Airline Type	0.1235	−0.0005	0.0556	1		
Review Valence	−0.0769	0.0381	0.1068	−0.0253	1	
Reviewer Expertise	0.2619	−0.0011	0.2397	0.0334	0.0875	1

**Table 6:** Examination of the variance inflation factors (VIFs).

**Variable**	**VIF**	**1/VIF**
Negative Safety Perception	1	0.997450
Review Modality	1.07	0.931619
Airline Type	1	0.995396
Review Valence	1.07	0.938043
Reviewer Expertise	1.02	0.982242

Online review usefulness in research is a non-negative count variable and may suffer from data dispersion, which is one of the common problems in review usefulness research (Pavlou and Dimoka, 2006; Lee et al., 2017). Due to the skewed distribution of the dependent variable, if we still use the standard multiple linear regression model for empirical analysis, it will lead to estimation bias (Zhou and Guo, 2017). For the above reasons, we should choose Poisson regression or negative binomial regression for empirical analysis that can effectively correct data dispersion problems (Yang et al., 2021). Poisson regression requires that the variance of the dependent variable is close to its mean, which means that the data need to satisfy the requirement of equal discrete distribution, but when the data are excessively discrete, the variance and mean of the dependent variable differ significantly, in which case, the choice of negative binomial regression should be considered, which allows a more effective fit to excessively discrete data (Lin et al., 2009). According to Table 4, the mean value of the dependent variable is 1.4632 and the standard deviation is 1.7739, and it can be found that the mean and variance of the dependent variable vary widely, so we choose negative binomial regression for testing the constructed model. Combining the proposed research hypothesis and the theoretical framework, the constructed model is defined as follows:

$\begin{array}{cc} \begin{array}{l} Review\\ Usefulness \end{array} & \begin{array}{c} =\beta_{0}+\beta_{1}\left(\mathrm{Negative}\;SafetyPerception_{i}\right)\\ +\beta_{2}\;\left(ReviewModality_{i}\right)+\beta_{3}\left(AirlineType_{i}\right)\\ +\beta_{4}\;\left(Negative\;SafetyPerception_{i}\times ReviewModality_{i}\right)\\ +\beta_{5}\left(Negative\;SafetyPerception_{i}\times AirlineType_{i}\right)\\ +\beta_{6}\left(ReviewValence_{i}\right)+\beta_{7}\left(ReviewExpertise_{j}\right)+\varepsilon_{i,j} \end{array} \end{array}$

where *β_0_* is the intercept term, *β_i_* (i = 1,2…7) is the regression coefficient, *i* represents the reviewer, j represents the reviewer, and *ε_i,j_* is the residual term.

We performed negative binomial regression on the constructed model through R software to analyze the direct effect of negative safety perception on online review usefulness, as well as the moderating effect of review modality and airline type on the relationship between negative safety perception and review usefulness, so as to verify whether our hypothesis holds. The results of the negative binomial regression are shown in Table 7. Table 7 reveals the regression results of 5 models in total. Among them, model 1 only contains control variables, which is used as the benchmark model, and independent variables are added to model 2 to test the main effect of negative safety perception on review usefulness. In Model 3 and Model 4, review modality and airline type and their interaction terms with independent variables are added to test the moderating effect of review modality and airline type on the main effect. Model 5 is the full model, including all variables. According to the regression results in Table 7, the Likelihood-ratio test of alpha equal to 0 is statistically significant (*p* < 0.001) from Model 1 to Model 5, which proves that the data does have the problem of overdispersion from another angle (Mariani and Borghi, 2021), and further verifies the rationality of choosing negative binomial regression.

**Table 7:** Negative binomial regression results.

**Variables**	**Model1**	**Model2**	**Model3**	**Model4**	**Model5**
***Direct Effects***					
Negative Safety Perception		−0.968^***^	−1.002^***^	−1.085^***^	−1.096^***^
		(−8.49)	(−8.79)	(−6.69)	(−6.77)
***Moderating Effects***					
Review Modality			0.456^***^		0.257^**^
			(3.73)		(2.16)
Airline Type				0.594^***^	0.588^***^
				(17.48)	(17.20)
Negative Safety Perception × Review Modality			1.398^*^		0.341
			(1.67)		(0.41)
Negative Safety Perception × Airline Type				1.484^***^	1.464^***^
				(6.17)	(6.07)
***Control Variables***					
Review Valence	0.088^***^	0.051^***^	0.045^***^	−0.039^***^	−0.042^***^
	(12.27)	(5.95)	(5.20)	(−3.76)	(−4.01)
Reviewer Expertise	0.000013^***^	0.000013^***^	0.000011^***^	0.000012^***^	0.000011^***^
	(11.84)	(12.30)	(9.69)	(13.01)	(10.69)
Observations	3,124	3,124	3,124	3,124	3,124
Log-likelihood	−4,923	−4,889	−4,883	−4,735	−4,732
LR chi2	353.9	432.7	459.8	859.6	872.3
Prob>chi2	0.000	0.000	0.000	0.000	0.000
LR test of alpha = 0	171.41	169.13	150.30	108.93	102.63
Prob> = chibar2	0.000	0.000	0.000	0.000	0.000

*N* = 3,124. Z-values are shown in parentheses.

*** *p* < 0.01;

** *p *< 0.05;

* *p* < 0.1.

As shown in Model 1, it can be seen that review valence (*β* = 0.088, *p* < 0.01) and reviewer expertise (*β* = 0.000013, *p* < 0.01) in the control variables both have significantly positive effects on the usefulness of online reviews, and the significant effects are still maintained after adding independent variables and interaction terms in Model 2-Model 5.

Hypothesis 1 proposes that negative safety perception has a significantly negative impact on review usefulness, that is, the more negative the safety perception of potential air passengers is, the more useful the reviews are considered. According to Model 2, the regression coefficient between negative safety perception and the usefulness of online reviews is significantly negative (*β* = −0.968, *p* < 0.01). Therefore, hypothesis 1 is supported.

After examining the main effect of Model 2, we also examined the moderating effect in Model 3 and 4. Model 3 analyzed the moderating effect of review modality, and the results indicate that the regression coefficient of the interaction term between negative safety perception and review modality is significant (*β* = 1.398, *p* < 0.1), while Hypothesis 2a assumes that online review modality significantly moderates the relationship between safety perception and review usefulness, therefore, Hypothesis 2a is supported.

Hypothesis 3a estimates that airline type plays a significant moderating role in the relationship between negative safety perception and review usefulness, while Model 4 analyzed the moderating effect of airline type on the main effect, the results illustrate that the regression coefficient of the interaction term between negative safety perception and airline type is significant (*β* = 1.484, *p* < 0.01). Therefore, Hypothesis 3a is supported.

**Figure 3:**
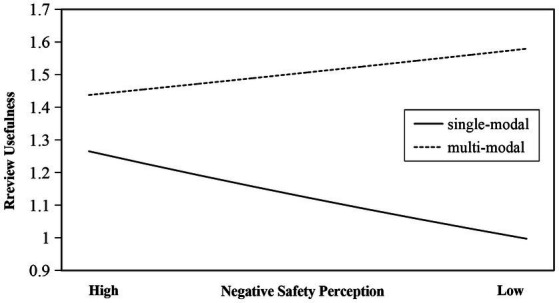
Moderating effect of review modality.

**Figure 4:**
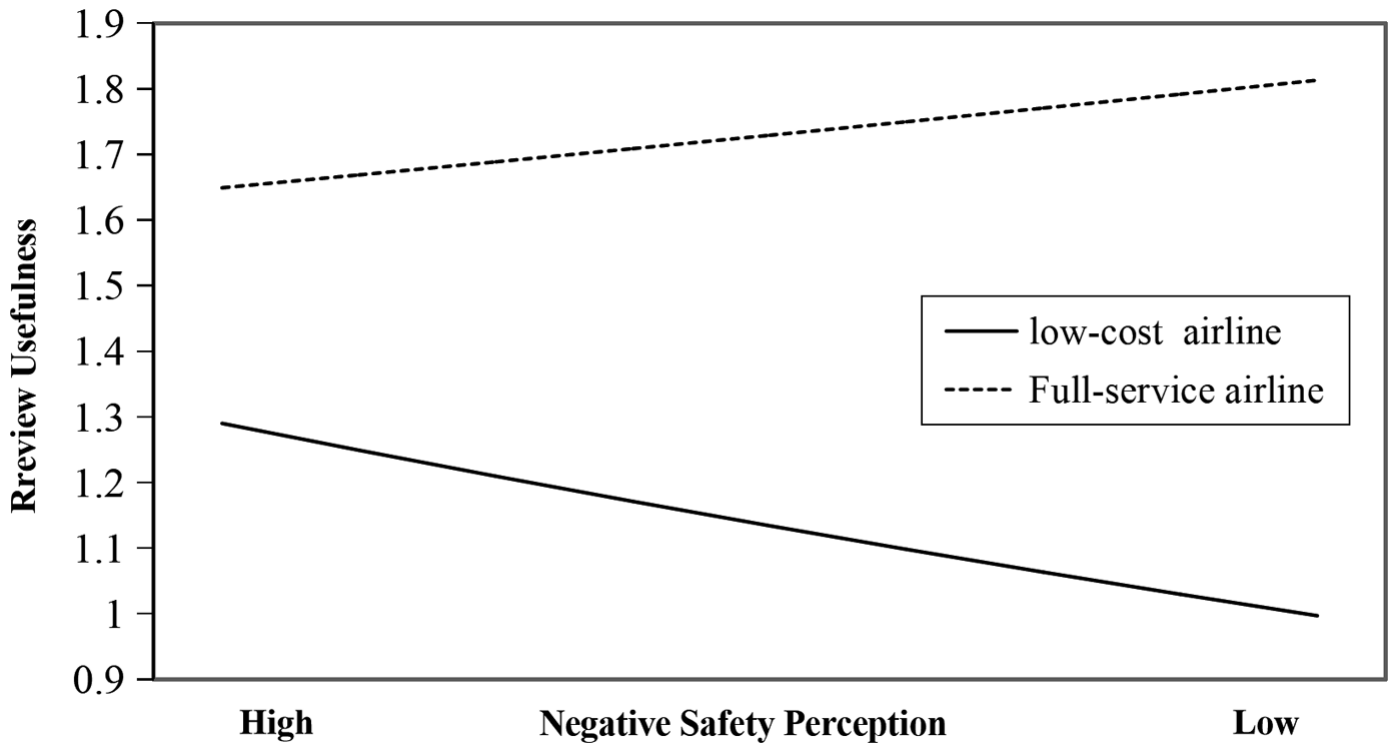
Moderating effect of airline type.

Figures 3, 4 present the plots of moderating effects for review modality and airline type. It can be seen from Figure 3 that when online reviews are multimodal, negative safety perception has a positive effect on review usefulness; however, when online reviews are single-modal, negative safety perception has a negative effect on review usefulness. That is, compared with single-modal reviews, multimodal reviews will positively influence the negative relationship between negative safety perception and review usefulness. We can also argue that multimodal reviews can weaken the negative impact of negative safety perception on review usefulness. This is absolutely inconsistent with Hypothesis 2b, so Hypothesis 2b is not supported.

As shown in Figure 4 that when online reviews come from full-service airlines, negative safety perception has a positive effect on review usefulness; when online reviews come from low-cost airlines, negative safety perception has a negative effect on review usefulness. Compared with low-cost airlines, full-service airlines positively affect the negative relationship between negative safety perception and review usefulness, in other words, full-service airlines can attenuate the negative effect of negative safety perception on review usefulness. This result is consistent with Hypothesis 3b, so Hypothesis 3b is supported.

In Model 5, except that the regression coefficient of the interaction term between negative safety perception and review modality is not significant, the coefficient direction and significance level of all other variables are in strong consistency with Model 2-Model 4, which proves that our negative binomial regression results are relatively robust. Table 8 summarizes our hypothesis testing results.

**Table 8:** Hypothesis testing results.

**Hypothesis**		**Result**
H1	Negative safety perception significantly and negatively affects review usefulness, i.e., extreme negative safety perception provides higher review usefulness for potential air passengers.	Support
H2a	The online review modality significantly moderates the relationship between negative safety perception and review usefulness.	Support
H2b	Compared with single-modality, multimodal reviews negatively moderate the relationship between negative safety perception and review usefulness, i.e., multimodal reviews reinforce the negative effect of negative safety perception on review usefulness.	Refuse
H3a	Airline type significantly moderates the relationship between negative safety perception and review usefulness.	Support
H3b	Compared with low-cost airlines, full-service airlines positively moderate the relationship between negative safety perception and review usefulness, i.e., full-service airlines attenuate the negative effect of negative safety perception on review usefulness.	Support

## Conclusion and discussion

### Conclusion

The outbreak and the continued spread of COVID-19 have caused a huge impact on the aviation industry, which seriously threatens the health and safety of air passengers. To reduce the likelihood of contracting the coronavirus, air passengers are forced to choose airlines that offer safe precautions based on online reviews. Therefore, this study seeks to explore whether negative e-WOM related to epidemic safety would make online reviews more useful. To achieve this goal, we constructed a research model based on prospect theory and protection motivation theory. From the perspective of “safety perception,” sentiment analysis is used to analyze the impact of potential air passengers’ negative safety perception on the usefulness of online reviews, the moderating roles of review modality and airline type on this effect are also considered. We empirically examined the research model with a large amount of real-world data collected from TripAdvisor, and the results reveal several exciting findings.

Firstly, our study examines the combined effects of specific review content (safety perception) and specific sentiment (negative sentiment) on the usefulness of online reviews. The findings suggest that reviews providing negative safety perception to potential air passengers are more useful. This is because, for potential passengers who have air travel needs and need to make airline choices during the epidemic, the negative safety perception obtained from online reviews can help them identify and avoid those high-risk airlines with poor epidemic prevention and control measures, thereby minimizing the safety risk of contracting the virus during air travel. The loss aversion property of prospect theory (Nicolau, 2011) provides theoretical support for the conclusion of this study. The findings can also support previous research findings (Cao et al., 2011; Fang et al., 2016; Lee et al., 2017; Li et al., 2020). However, some scholars have come to the opposite conclusions that positive e-WOM is more convincing (Doh and Hwang, 2009; Zhang et al., 2021), and the real characteristics of neutral emotions will make the reviews more credible (Filieri et al., 2018). However, in the particular context of a global threat of the epidemic, people are much more willing to avoid risks than to take them (Nicolau, 2011). Negative safety perception are thus likely to be a decisive factor in judging the usefulness of a review compared with the positive and neutral affective factors influencing the review usefulness (Seabra et al., 2013).

Furthermore, our study shows that the modality of online reviews can significantly moderate the relationship between negative safety perception and review usefulness. But contrary to our hypothesis, multimodal reviews instead attenuate the negative effect of negative safety perception on online review usefulness compared to single-modal reviews, which is also inconsistent with the current research findings. The dual coding theory of cognitive psychology argues that presenting information in both visual and textual forms can reduce cognitive load and improve the accuracy of information recognition (Wang and Chen, 2018; Ma et al., 2020),which proves that online reviews combined with images and texts are more likely to obtain review usefulness votes. Based on the above existing research results, we have reason to believe that multimodal online reviews can strengthen the negative impact of negative safety perception on review usefulness. But interestingly, in our findings, multimodal reviews positively affect the negative relationship between negative safety perception and review usefulness. We attempt to rationalize the research findings from the perspective of e-WOM communication motivations. The motivations for e-WOM communication are categorized as social interaction, economic incentives, concern for others, and self-realization. There is an existing research consensus that more negative reviews are more useful, but this consensus is mostly based on studies of single-modal text reviews. With the increase in review modalities, the motivation to post multimodal reviews has become not only to help potential consumers improve their decision-making quality by expressing emotions and stating facts but also to achieve social interaction (Lai et al., 2021) and self-worth enhancement (Cheung and Lee, 2012). Because adding pictures will provide more vivid information in the reviews, it will be more easily promoted by third-party platforms, which means that more platform users will view and follow (Zhu et al., 2020), so that reviewers will gain inner satisfaction and Pleasure (Cheung and Lee, 2012). Therefore, users who post multimodal reviews will deliberately reduce the impact of their extreme negative sentiments on the content of the reviews. This behavior makes the review content for safety perception more objective with the aim of gaining more platform promotion and social interaction (Lai et al., 2021), which is why multimodal reviews weaken the negative impact of negative safety perception on review usefulness.

Finally, we demonstrate that the impact of negative safety perception on online review usefulness is significantly moderated by airline type, and full-service airlines attenuate the negative impact of negative safety perception on review usefulness compared with low-cost airlines. This is consistent with our research hypothesis, and previous research results also provide support for our findings. Passengers will be affected by price and service quality when choosing different types of airlines (Kim and Lee, 2011). According to social exchange theory (Emerson, 1976), air passengers are willing to pay higher prices in exchange for better safety service quality of full-service airlines. This means that full-service airlines are considered to be capable of taking more effective epidemic prevention and control measures than low-cost airlines. It is this stereotype that increases people’s tolerance for full-service airline service failures and eases safety concerns for full-service airlines, thereby positively moderating the negative impact of negative safety perception on review usefulness.

### Theoretical implications

The theoretical implications of this study are mainly reflected in the following aspects. First, we extend previous research on factors influencing the usefulness of online reviews to the aviation industry. Superimposed on the background of the epidemic, according to the loss aversion characteristics of prospect theory, the current air passengers’ fear and safety demands of the epidemic are accurately captured, which confirms the significant impact of potential air passengers’ safety perception on the airline choices. Different from the risk aversion perspectives of prospect theory and protection motivation theory, this theoretical model pioneers the exploration of influencing factors of online review usefulness from the perspective of safety perception, and develops traditional prospect theory and protection motivation theory.

Second, our research applies big data sentiment analysis technology to the e-WOM management of airlines, which derives a new research direction from the traditional e-WOM research in the aviation industry. This study utilizes big data sentiment analysis technology to further focus on the negative e-WOM of air passengers’ safety perception, and validates that negative e-WOM has more powerful effects on review usefulness in the aviation domain.

Finally, unlike previous single-modal e-WOM studies based solely on textual reviews, this study also examines the influence of multimodal data on the relationship between negative safety perception and review usefulness. Although our findings are somewhat inconsistent with previous studies suggesting that multimodal data can improve review usefulness (Zhao et al., 2019; Zhang et al., 2021), we rationalize the interpretation from the perspective of social motivation for e-WOM propagation (Zhu et al., 2020; Lai et al., 2021), which further enriches research on the relationship between review modality, review sentiment, and review usefulness. It is worth mentioning that this study also found that air passengers have stereotypes about different types of airlines. Specifically, they have higher high-quality service expectations and service failure tolerance for full-service airlines, and this asymmetry also significantly affects the relationship between negative safety perception and review usefulness.

### Practical implications

This study also provides some managerial implications. First, the conclusions of this study provide a new reference for potential air passengers to identify useful online reviews, which can help reduce the safety risks of choosing airlines through online travel platforms during the epidemic. Our research reveals the impact of air passengers’ negative safety perception on the usefulness of online reviews, suggesting that air passengers should pay more attention to negative reviews that contain safety information on epidemic prevention and control when browsing airline online reviews. The study also demonstrates that multimodal reviews and full-service airlines can moderate the impact of negative safety perception on review usefulness, suggesting that air passengers should also consider review modality and airline type in the process of acquiring negative safety perception and judging whether online reviews are useful.

Secondly, from the perspective of airlines, the conclusions of this study have important reference value for airlines’ e-WOM management and improvement, and provide new insights into how to promote the rapid recovery of the aviation industry during the epidemic. Airlines should attach great importance to the e-WOM of online travel platforms, especially the negative e-WOM related to the safety of epidemic prevention and control because the safety concerns of air passengers about contracting the virus during travel are the main pain points that prevent airline performance from recovering. Therefore, airlines should clarify the current safety hazards based on the negative safety perception reviews released by passengers, and timely improve epidemic safety measures to provide passengers with more reliable safety services. We encourage airlines to view and respond to negative reviews related to safety perception in a timely manner (Chen et al., 2021), to make up for the failure of safety services by increasing interaction and rebuild the sense of safety and trust of air passengers. It should be noted that, due to people’s stereotypes (Xu et al., 2019), low-cost airlines must invest more energy in e-WOM management than full-service airlines, and need to take more active epidemic safety measures to eliminate air passengers’ safety concerns.

Finally, our research also provides improvement guidelines for the operation of third-party online travel platforms. In response to the safety threats caused by the epidemic, online travel platforms should use advanced sentiment analysis algorithms to identify the sentiment polarity of reviews, and set up separate review screening labels related to epidemic safety in combination with the review modality and airline type. These measures allow air passengers to quickly and accurately identify useful reviews, avoid information overload, and reduce online decision-making costs and safety risks. At the same time, a comment reminder function is needed for airlines to remind the latest negative safety reviews in a targeted manner (Chen et al., 2021), so that airlines can improve epidemic safety measures in a precise and timely manner, and promote the recovery of airline performance through e-WOM management.

### Limitations and future research directions

This study still has certain limitations and needs to be further improved in future research. First of all, this study only selects the English review data of a single online travel platform, and does not collect review information in other languages such as Chinese, resulting in a single sample source and sample data, which may bias the results of sentiment analysis. In future research, we consider collecting multilingual review data from multiple third-party online platforms to verify the generality of the research. Second, since our chosen TripAdvisor platform only allows text and image reviews, the review modalities involved in the study are still relatively single. In future research, we try to add more diverse modalities such as the video reviews containing audio information to enrich the study of multimodal reviews. Third, this study only selected Textblob as a sentiment analysis method and did not strictly evaluate the accuracy of sentiment analysis. Future research plans to select three or more sentiment analysis techniques, conduct a horizontal comparison of the effects of sentiment analysis, and select the analysis method with the best accuracy for the next empirical test.

## Data availability statement

The original contributions presented in the study are included in the article/supplementary material; further inquiries can be directed to the corresponding author.

## Author contributions

SB and DY: conceptualization. MY and DY: methodology. RT: formal analysis. DY: writing-original draft. HH and JZ: data curation and software. DY and PH: writing-review and editing. MY: supervision. SB: project administration and funding acquisition. All authors contributed to the article and approved the submitted version.

## Funding

This research was supported by the National Natural Science Foundation of China (Project 71671054) and the reform and develop high-level talent projects in local universities supported by the central government (2020GSP13).

## Conflict of interest

The authors declare that the research was conducted in the absence of any commercial or financial relationships that could be construed as a potential conflict of interest.

## Publisher’s note

All claims expressed in this article are solely those of the authors and do not necessarily represent those of their affiliated organizations, or those of the publisher, the editors and the reviewers. Any product that may be evaluated in this article, or claim that may be made by its manufacturer, is not guaranteed or endorsed by the publisher.
